# Mutant IDH1 is required for IDH1 mutated tumor cell growth

**DOI:** 10.18632/oncotarget.577

**Published:** 2012-08-09

**Authors:** Genglin Jin, Christopher J. Pirozzi, Lee H. Chen, Giselle Y. Lopez, Christopher G. Duncan, Jie Feng, Ivan Spasojevic, Darell D. Bigner, Yiping He, Hai Yan

**Affiliations:** ^1^ The Preston Robert Tisch Brain Tumor Center, The Pediatric Brain Tumor Foundation Institute, and The Department of Pathology; ^2^ The Clinical Pharmacology Laboratory, Duke Cancer Institute and Department of Medicine/Oncology, Duke University Medical Center, Durham, North Carolina, USA

**Keywords:** IDH1, cell survival

## Abstract

Frequent somatic hotspot mutations in isocitrate dehydrogenase 1 (IDH1) have been identified in gliomas, acute myeloid leukemias, chondrosarcomas, and other cancers, providing a likely avenue for targeted cancer therapy. However, whether mutant IDH1 protein is required for maintaining IDH1 mutated tumor cell growth remains unknown. Here, using a genetically engineered inducible system, we report that selective suppression of endogenous mutant IDH1 expression in HT1080, a fibrosarcoma cell line with a native IDH1^*R132C*^ heterozygous mutation, significantly inhibits cell proliferation and decreases clonogenic potential. Our findings offer insights into changes that may contribute to the inhibition of cell proliferation and offer a strong preclinical rationale for utilizing mutant IDH1 as a valid therapeutic target.

## INTRODUCTION

Genome-wide mutational analyses have revealed somatic mutations in the cytosolic NADP+-dependent isocitrate dehydrogenase 1 (*IDH1*) in 12% of glioblastoma multiforme (GBM) patients [1]. Further studies identified mutations in *IDH1* affecting amino acid 132 in over 70% of WHO grade II and III astrocytomas, oligodendrogliomas, and secondary glioblastomas [2-4]. Less frequently, mutations have been identified in the homologous mitochondria-associated *IDH2* at the analogous amino acid residue (R172) [3, 5]. Following the discovery of these mutations at a surprisingly high frequency in low grade gliomas, *IDH1* and *IDH2* mutations were subsequently observed in several other cancers and syndromes, including acute myeloid leukemia [6-8], preleukemic clonal malignancies [9], central and periosteal cartilaginous tumors [10], intrahepatic origin cholangiocarcinomas [11], Ollier disease and Maffucci syndrome [12, 13]. More rarely, *IDH1* and *IDH2* mutations have been reported in prostate cancer [14], B-acute lymphoblastic leukemia [14], colorectal cancer [15] and melanoma [16]. Additionally, *IDH2* germline mutations were detected in some patients with D-2-hydroxyglutaric aciduria [17]. The presence of mutations in *IDH1* and *IDH2* early in disease pathogenesis in malignant gliomas, myeloid malignancies, and cartilaginous tumors underscores the magnitude of the role of *IDH1* and *IDH2* mutations in tumorigenesis [18, 19].

Mutations in the active site of IDH1 (R132) and IDH2 (R172) impair the conversion of isocitrate to α-ketoglutarate (αKG) [3, 20]. Metabolic profiling has identified a neomorphic activity in the presence of the mutant enzyme, namely, generation of a novel onco-metabolite, D-2-hydroxyglutarate (D-2HG) [4, 21]. Additionally, elevated D-2HG levels were found in AML and brain tumor patients with *IDH1* and *IDH2* mutations [21-23]. Nonetheless, the ramifications of these mutations remain largely unclear, and research is only recently starting to uncover some of the potential roles these mutations may play. D-2HG is structurally similar to αKG, and thus may act through the inhibition of enzymes requiring αKG. Large-scale analyses of genome-wide DNA methylation patterns in human gliomas found that *IDH1/2* mutations were associated with a highly specific DNA methylation profile [24-26]. It has been postulated that the changes in methylation may be due to D-2HG-mediated inhibition of multiple αKG-dependent dioxygenases, including the Tet oncogene family (TET) of 5-methylcytosine (5mC) hydroxylases and histone demethylases, resulting in increased histone methylation and decreased 5-hydroxymethylcytosine (5hmC) modifications [27, 28]. Further studies confirmed that mutant IDH1 impairs histone demethylation and is sufficient to establish the glioma hypermethylator phenotype, resulting in a block to cell differentiation [29].

The high frequency of *IDH1* and *IDH2* mutations in certain cancers, and the specificity of the genetic alterations to a limited set of amino acid residues, makes the mutant IDH1 or IDH2 product a tempting target for therapy. However, the role and contribution of mutant *IDH1* or *IDH2* in tumor initiation and/or maintenance remains largely unresolved, with findings from different groups yielding conflicting results. While one group reported increased growth *in vivo* in a melanoma cell line transfected with mutant IDH1 [30], another group reported a decrease in proliferation in glioma cell lines both *in vitro* and *in vivo* in the presence of mutant IDH1 [31]. However, both studies utilized cancer cell lines with wild type IDH1 into which the mutant IDH1 was introduced. To address this issue, we studied HT1080, a fibrosarcoma cell line carrying a native *IDH1^R132C^* heterozygous mutation, and found that selectively knocking down mutant IDH1 inhibited cell growth. The critical role the mutant allele is playing in cell survival strongly infers that mutant IDH1 can be used as a practical therapeutic target against *IDH1* mutated cancers.

## RESULTS

### Knockdown of both wild type and mutant IDH1 expression inhibits HT1080 cell proliferation

**Figure 1:**
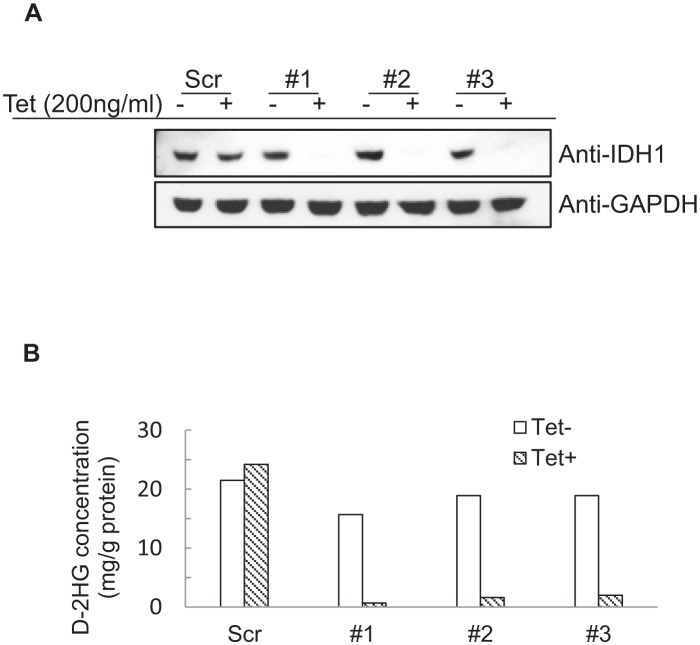
Establishment of IDH1 inducible knockdown HT1080 cell lines A, Western blot detection of IDH1 in three different clones following treatment with 200ng/mL tetracycline for 96 hours. B, D-2HG concentration in cell lysates following treatment with 200ng/mL tetracycline for 96 hours.

**Figure 2:**
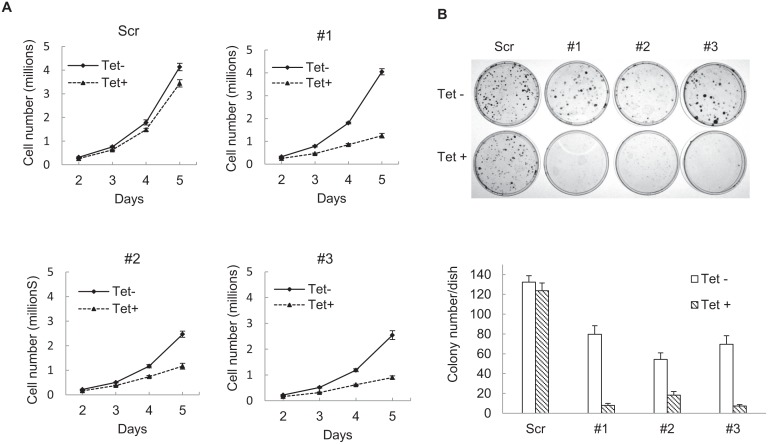
Knocking down IDH1 expression inhibits HT1080 cell growth A, 50,000 cells were seeded into 100mm cell culture dishes, with 0 or 200ng/mL tetracycline. Cells were collected and counted daily for five days. Studies were done with biological triplicates. B, 300 cells were plated into 60mm cell culture dishes. After 8-10 days, cell colonies were stained with 0.05% crystal violet. Every treatment was completed in biological triplicate. Representative cell culture dishes are shown.

To assess whether mutant and wild type IDH1 contribute to *IDH1* mutated tumor cell growth, we created an IDH1 inducible knockdown cell line utilizing the fibrosarcoma cell line, HT1080 which harbors a heterozygous *IDH1^R132C^* mutation. Lentivirus encoding a tetracycline-inducible shRNA targeting the 3’ noncoding mRNA of *IDH1* was used to generate IDH1 inducible knockdown cell lines. As shown in Figure 1, treatment with 200ng/ml tetracycline for 96 hours efficiently knocked down both wild type and mutant IDH1 expression in three different IDH1 inducible knockdown HT1080 clones by the shRNA. Decreased IDH1 expression led to a ten-fold decrease in levels of D-2HG (Figure 1B). We assessed the proliferative activities of the clones and observed a substantial decrease (62.12% ± 6.93%) in proliferation following IDH1 knockdown by tetracycline (Figure 2A). Colony formation assays confirmed our finding that HT1080 cells exhibited decreased clonogenic survival (81.9% ± 13.54%) upon IDH1 knockdown by tetracycline. Additionally, compared to the scrambled shRNA control, colonies that did form following IDH1 knockdown were smaller in size (Figure 2B).

### Mutant IDH1^R132C^ is critical for tumor cell proliferation

**Figure 3:**
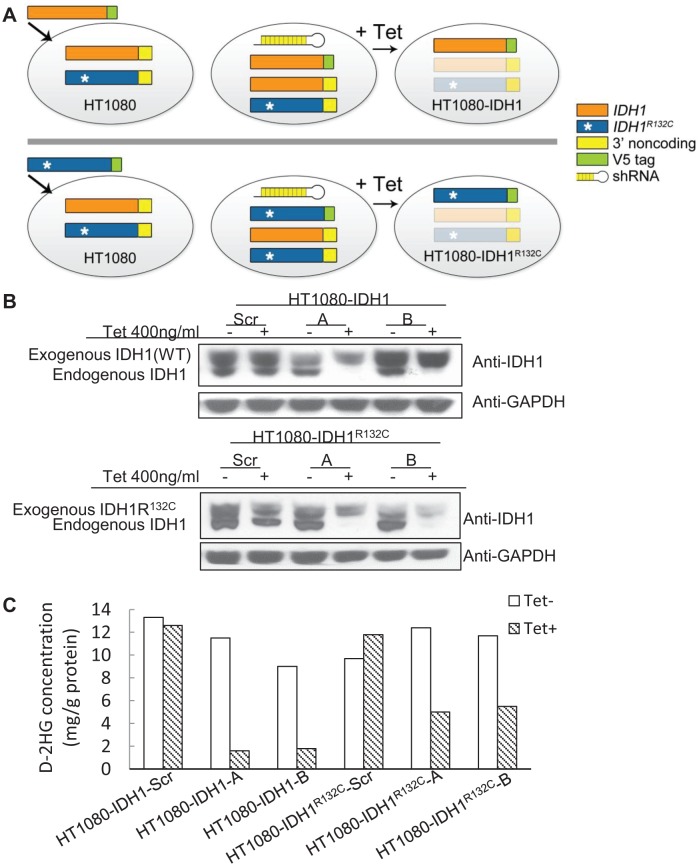
Generation of cell lines with inducible knockdown of mutant or wild type IDH1 A, Strategy for generating cell lines with inducible knockdown of mutant or wild type IDH1. IDH1 or IDH1^R132C^ full-length cDNA containing a V5 tag and lacking the 3’ non-coding sequence was infected into HT1080 cells and selected for to generate stable cell lines that exogenously express IDH1 or IDH1^R132C^. A tetracycline-inducible knockdown vector containing a shRNA sequence specific for the IDH1 3’ non-coding sequence was used to infect the stable cell lines that exogenously express IDH1 or IDH1^R132C^. Following treatment with tetracycline, the endogenous pools of wild type and mutant IDH1 were ablated, while expression of exogenous IDH1 or IDH1^R132C^ remained intact; thereby allowing for the study of the effects of wild type or mutant IDH1 knockdown. B, Cell lines with knockdown of either wild type IDH1 or IDH1^R132C^ as identified by Western blot following a 96 hour treatment with tetracycline. C, D-2HG concentration in cell lysates following 200ng/mL tetracycline treatment for 96 hours.

**Figure 4:**
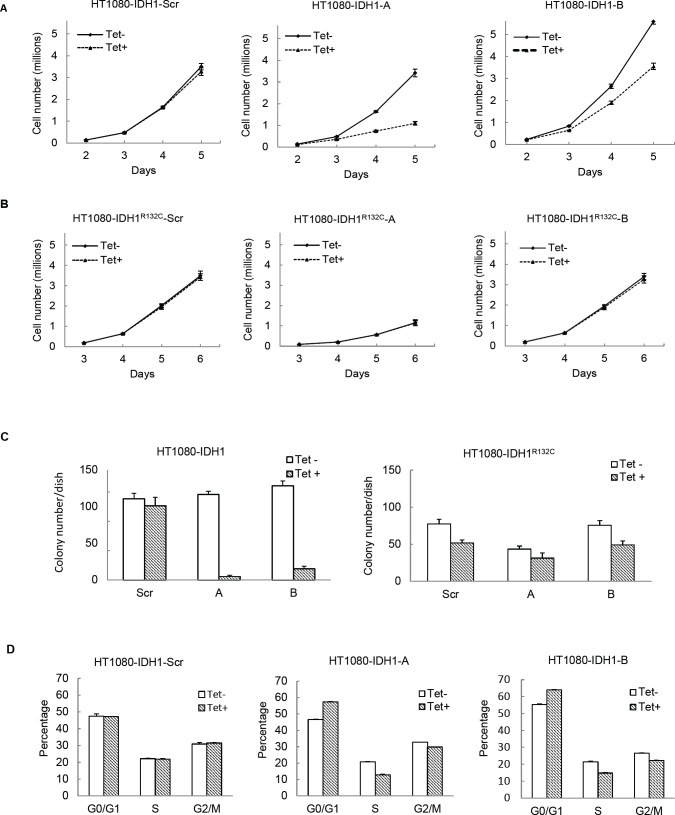
Knocking down mutant IDH1^R132C^ inhibits cell growth in vitro A, 50,000 cells with exogenous wild type IDH1 expression and inducible knockdown of endogenous IDH1 were seeded in 100mm cell culture dishes in triplicate and treated with 0 or 200ng/mL tetracycline. Cells were collected and counted daily for 5 days. Error bars represent one standard deviation. B, 50,000 cells with exogenous *IDH1^R132C^* expression and inducible knockdown of endogenous IDH1 were seeded in 100mm cell culture dishes in triplicate and treated with 0 or 200ng/mL tetracycline. Cells were collected and counted daily for 6 days. C, 300 cells were plated in 60mm cell culture dishes in triplicate. After 8-10 days treatment, cell colonies were stained with 0.05% crystal violet and counted. D, Indicated cell lines were treated with 0 or 200ng/mL for four days in triplicate and fixed. Cell cycle analysis was completed via FACS.

The above shRNA used is unable to distinguish between mutant or wild type *IDH1*. To further reveal the role of mutant IDH1 in cell proliferation, we established multiple cell lines in which we specifically expressed either mutant or wild type IDH1 while knocking down endogenous IDH1 and IDH1^R132C^ in HT1080 by tetracycline induction. This allowed for the simulation of a specific knockdown of either wild type or mutant IDH1, depending on which was being exogenously expressed. First, to generate stable cell lines that exogenously express either wild type IDH1 or mutant IDH1^R132C^, we introduced full-length *IDH1* or *IDH1^R132C^* cDNA missing the 3’ noncoding region, and with the addition of a V5 tag, to HT1080 cells. Second, we infected these same cell lines with lentivirus containing an inducible shRNA targeting the *IDH1* 3’ noncoding region. This led to the inducible knockdown of both endogenous wild type IDH1 and endogenous IDH1^R132C^, but left the exogenous, lentiviral-mediated expression of IDH1 or IDH1^R132C^ intact, subsequently allowing for the simulation of specific knockdown of IDH1^R132C^ or IDH1, respectively (Figure 3A). Western blot assay revealed that tetracycline decreased endogenous IDH1 expression efficiently. Exogenously expressed IDH1 or IDH1^R132C^ is larger than endogenous IDH1 due to the V5 tag and allowed for visualization of endogenous IDH1 knockdown (Figure 3B). Knocking down endogenous IDH1 expression also decreased the D-2HG concentration in HT1080 cells (Figure 3C). Cell proliferation assays revealed that knocking down endogenous wild type and mutant *IDH1* expression while maintaining exogenous wild type *IDH1* expression in HT1080 inhibited cell growth (52.20% ± 15.73%). On the contrary, knocking down endogenous wild type and mutant IDH1 while maintaning exogenous IDH1^R132C^ expression in HT1080 had a minimal effect on cell proliferation (Figure 4A,B). This observation was further confirmed by colony formation assay (Figure 4C). Fluorescence Activated Cell Sorting (FACS) cell cycle analysis revealed that knocking down endogenous mutant and wild type IDH1 expression while maintaining exogenous wild type IDH1 expression in HT1080 arrested the cells at the G_0_/G_1_ phase (Figure 4D).

## DISCUSSION

Thus far, studies that have sought to determine the contribution of mutant IDH1 in tumor survival and proliferation have conflicted, making the potential of mutant IDH1 as a therapeutic target difficult to defend. Here, we describe the generation and use of a biologically relevant cell line that simulates the knockdown of either IDH1 or IDH1^R132C^. Our system allows us to dissect the relative contribution of wild type and mutant IDH1 in the fibrosarcoma cell line, HT1080 which harbors a heterozygous R132C mutation in *IDH1*. This system offers insights into the role that mutant IDH1 plays in tumor cell survival and maintenance. Knockdown of mutant IDH1 in the HT1080 tumor cell line confers significant decreases in cellular proliferation, decreases in the onco-metabolite D-2HG, overall decreases in clonogenic potential, as well as an induction of a G_0_/G_1_ cell cycle arrest; thereby suggesting that depletion of mutant IDH1 from an IDH1 mutant tumor cell line decreases the tumorigenic potential of these cells. Because of the specificity of mutant IDH1 to tumor cells and its absence in the surrounding parenchyma, drugs specifically designed to disrupt mutant IDH1 may kill tumor cells with *IDH1* mutations while sparing normal tissues. This, together with our findings that mutant IDH1 is critical for cell survival, offers convincing preclinical rationale for utilizing mutant IDH1 as a therapeutic target.

## MATERIALS AND METHODS

### Virus

To express the *IDH1* and *IDH1^R132C^* transgenes in cells, *IDH1* and *IDH1^R132C^* missing the 3’ noncoding sequence were cloned into pLenti6.2/V5 (Invitrogen) following the manufacturer's instructions. To generate the IDH1 inducible knockdown vector, the stuffer DNA was removed from pLKO-Tet-On (Novartis) by AgeI/EcoRI digest and replaced with double-stranded oligos encoding the *IDH1* specific shRNA sequences (targeting the *IDH1* 3’ noncoding sequence) with AgeI/EcoRI sites. Viruses were created using these constructs in 293FT cells, following the manufacturer's instructions.

### Cell lines

The fibrosarcoma cell line HT1080 was generated from a fibrosarcoma biopsy taken from a 35 year-old male patient and was provided by Dr. Todd Waldman of Georgetown University [32]. HT1080 cells were cultured in DMEM (Gibco, Cat. No. 11995) with 10% FBS. To establish IDH1 inducible knockdown cell lines, HT1080 cells were infected with lentivirus encoding a tetracycline-inducible shRNA targeting the 3’ noncoding mRNA of *IDH1* and selected for with 0.8μg/ml puromycin for three weeks. The derived clones, HT1080-shRNA clone #1, #2, and #3 were used for subsequent knockdown studies. Lentivirus containing a tetracycline-inducible scrambled shRNA was used as a control.

To determine the contribution of wild type or mutant IDH1, *IDH1* or *IDH1^R132C^* full-length cDNA with a V5 tag and lacking the 3’ non-coding sequence was exogenously expressed in HT1080 cells. Cells were selected with 4 μg/ml blasticidin for three weeks. Infection with tetracycline-inducible shRNA targeting the 3’ noncoding mRNA of *IDH1* (the same lentivirus described above), depleted endogenous pools of IDH1 and IDH1^R132C^. Cells were selected with 0.8μg/ml puromycin for three weeks. Scrambled shRNA expressing lentivirus was used as a control. Tetracycline (Sigma, St. Louis, MO) was dissolved in PBS and used for shRNA induction.

### Western blot

Total protein was isolated from cultured monolayer cells with M-PER Mammalian Protein Extraction Reagent (Thermo Scientific). Protein concentrations were measured by comparing with Pre-Diluted Protein Assay standards (Thermo Scientific). After incubation at 95°C for five minutes, 50μg protein with same volume in 5% β-mercaptoethanol Laemmli Sample Buffer (BIO-RAD) was loaded onto SDS-PAGE and transferred electrophoretically to nitrocellulose membranes (BIO-RAD). Membranes were blocked with 5% milk in washing buffer (10 mmol Tris-HCI pH 7.5, 150 mmol NaCI, 0.05% Tween 20) at room temperature for two hours. Membranes were then incubated at 4°C with anti-IDHC (sc49996, Santa Cruz Biotechnology,Santa Cruz, CA 1:2000 dilution) or anti-GAPDH (sc25778, Santa Cruz Biotechnology, Santa Cruz, CA 1:10000 dilution) overnight at 4°C. Bands were detected using SuperSignal West-Femto Maximum Sensitivity Substrate (Thermo Scientific).

### Cell Proliferation and Clonogenic Assays

For cell proliferation studies, cells were seeded at a density of 50,000 per 100mm diameter dish and treated with 0 or 200 ng/ml tetracycline. After 5-6 days, cells were collected and counted. For clonogenic survival assays, cells were plated in 60mm culture dishes (300 cells/dish) and treated with 0 or 200 ng/mL tetracycline for 8-10 days. Cell colonies were stained with 0.05% crystal violet (Sigma) for one hour and then washed with water. Assays were completed in triplicate.

### Cell-cycle Analysis

Cells were induced with 0 or 200ng/mL tetracycline for four days, collected, fixed in 70% ice-cold ethanol for 24 hours, and treated with 20 μg/mL propidium iodide, 0.1% (v/v) Triton X-100, and 100 μg/mL DNase-free RNase A for 20 minutes. Samples were analyzed by BD FACSCalibur.
